# Risk Factors, Temporal Dependence, and Seasonality of Human Extended-Spectrum β-Lactamases-Producing *Escherichia coli* and *Klebsiella pneumoniae* Colonization in Malawi: A Longitudinal Model-Based Approach

**DOI:** 10.1093/cid/ciad117

**Published:** 2023-03-04

**Authors:** Melodie Sammarro, Barry Rowlingson, Derek Cocker, Kondwani Chidziwisano, Shevin T Jacob, Henry Kajumbula, Lawrence Mugisha, David Musoke, Rebecca Lester, Tracy Morse, Nicholas Feasey, Chris Jewell

**Affiliations:** Department of Clinical Sciences, Liverpool School of Tropical Medicine, Liverpool, United Kingdom; Centre for Health Informatics, Computing, and Statistics, Lancaster University, Lancaster, United Kingdom; Centre for Health Informatics, Computing, and Statistics, Lancaster University, Lancaster, United Kingdom; Department of Clinical Sciences, Liverpool School of Tropical Medicine, Liverpool, United Kingdom; Malawi-Liverpool-Wellcome Research Programme, Kamuzu University of Health Sciences, Blantyre, Malawi; Centre for Water, Sanitation, Health and Appropriate Technology Development (WASHTED), Malawi University of Business and Applied Sciences (MUBAS), Blantyre, Malawi; Department of Civil and Environmental Engineering, University of Strathclyde, Glasgow, United Kingdom; Department of Clinical Sciences, Liverpool School of Tropical Medicine, Liverpool, United Kingdom; Global Health Security Department, Infectious Disease Institute, Makerere University, Kampala, Uganda; Department of Medical Microbiology, College of Health Sciences, Makerere University, Kampala, Uganda; College of Veterinary Medicine, Animal Resources and Biosecurity (COVAB), Makerere University, Kampala, Uganda; Conservation & Ecosystem Health Alliance, Kampala, Uganda; Department of Disease Control and Environmental Health, College of Health Sciences, Makerere University, Kampala, Uganda; Department of Clinical Sciences, Liverpool School of Tropical Medicine, Liverpool, United Kingdom; Malawi-Liverpool-Wellcome Research Programme, Kamuzu University of Health Sciences, Blantyre, Malawi; Centre for Water, Sanitation, Health and Appropriate Technology Development (WASHTED), Malawi University of Business and Applied Sciences (MUBAS), Blantyre, Malawi; Department of Civil and Environmental Engineering, University of Strathclyde, Glasgow, United Kingdom; Department of Clinical Sciences, Liverpool School of Tropical Medicine, Liverpool, United Kingdom; Malawi-Liverpool-Wellcome Research Programme, Kamuzu University of Health Sciences, Blantyre, Malawi; Centre for Health Informatics, Computing, and Statistics, Lancaster University, Lancaster, United Kingdom

**Keywords:** antimicrobial resistance, Africa, community

## Abstract

**Background:**

Sub-Saharan Africa has the highest estimated death rate attributable to antimicrobial resistance, especially from extended-spectrum β-lactamase-producing Enterobacterales (ESBL-E). However, the dynamics of human colonization in the community with ESBL-E are not well described. Inadequate water, sanitation, and hygiene infrastructure and associated behaviors are believed to play an important role in transmission of ESBL-E, and an improved understanding of the temporal dynamics of within-household transmission could help inform the design of future policies.

**Methods:**

In this 18-month study, using microbiological data and household surveys, we built a multivariable hierarchical harmonic logistic regression model to identify risk factors for colonization with ESBL-producing *Escherichia coli* and *Klebsiella pneumoniae*, reflecting household structure and temporal correlation of colonization status.

**Results:**

Being male was associated with a lower risk of colonization with ESBL-producing *E. coli* (odds ratio [OR], 0.786; credible interval [CrI], .678–.910), whereas the use of a tube well or a borehole was associated with an increased risk (OR, 1.550; CrI, 1.003–2.394). For ESBL-producing *K. pneumoniae*, recent antibiotic exposure increased risk of colonization (OR, 1.281; CrI, 1.049–1.565), whereas sharing plates decreased that risk (OR, 0.672; CrI, .460–.980). Finally, the temporal correlation range of 8 to 11 weeks provided evidence that within-household transmission occurs within this time frame.

**Conclusions:**

We describe different risks for colonization with different enteric bacterial species. Our findings suggest interventions to reduce transmission targeted at the household level need to focus on improving water, sanitation, and hygiene infrastructure and associated behaviors, whereas at the community level, they should focus on both environmental hygiene and antibiotic stewardship.

## Teaser

Water, sanitation, and hygiene (WASH) factors are important drivers of extended-spectrum β-lactamases (ESBL)-producing *Escherichia coli* transmission, with contaminated water and/or inappropriate infrastructure the likely mode of acquisition. In contrast, antimicrobial exposure appears as the key driver for ESBL-producing *Klebsiella pneumoniae*.

In 2015, the World Health Organization declared antimicrobial resistance (AMR) as a priority public health threat [1, 2]. The latest estimates showed that 4.95 million deaths were associated with bacterial AMR in 2019 [3]. The attributable death rate was highest in sub-Saharan Africa (sSA), where leading pathogens included *Klebsiella pneumoniae* and *Escherichia coli*; however, large gaps in data availability were noted [3]. Of particular note has been the rapid emergence of extended-spectrum β-lactamases (ESBL) in gram-negative bacteria [4]. Studies have shown that the prevalence of infections caused by ESBL-producing Enterobacterales (ESBL-E) in sSA is high, with prevalence varying between 5% and 84%, with a relationship to setting [5, 6]. A separate 2019 review found a median prevalence of resistance of 14.3% in *E. coli* and 46.7% in *Klebsiella* spp. in Eastern Africa [7]. Learning more about asymptomatic colonization, a key step before infection in vulnerable patient groups, is therefore crucial to prevent transmission, and consequently, reduce drug-resistant infections.

Before 2016, no studies described risk factors for colonization among healthy individuals in the community in sSA [5]. Antibiotic use, age, hospital admission, and income have since been identified in community-based studies [8–11]. However, most of them focused on a specific subset of the community; therefore, we cannot be confident these risk factors are generalizable across the population. Moreover, although it is believed that inadequacies in water, sanitation, and hygiene (WASH) infrastructure and associated behaviors play an important role in transmission of AMR bacteria [12, 13], risk factors related to WASH in this context are not well described.

To be able to reduce transmission of, and colonization by, AMR enteric bacteria in sSA, we need to explore the dynamics of colonization to tailor appropriate interventions; for example, the role of seasonality. A first step in understanding the temporal dynamics of within-household transmission is to determine how long a household is at risk of colonization once one member has been colonized. This will help inform the design of public health policies that interrupt transmission of AMR bacteria.

Here, we describe an analysis of an 18-month longitudinal cohort study using microbiological, household, and WASH surveys, where WASH refers to containment of human and animal feces, hygiene, and food hygiene. We fit a serially correlated generalized linear mixed model, exploring household, individual, and WASH risk factors for ESBL-E colonization in settings with different degrees of urbanization in Malawi.

## METHODS

The Drivers of Resistance in Uganda and Malawi (DRUM) consortium was an interdisciplinary consortium studying AMR transmission in a One Health setting, working across urban, peri-urban, and rural communities in Uganda and Malawi (www.drumconsortium.org). DRUM was a repeated-measures study in which individuals, clustered into households, were sampled at 4 timepoints over 6 months. Longitudinal human stool, animal and environmental sampling were used to isolate ESBL *E. coli* (ESBL-Ec) and ESBL *K. pneumoniae* (ESBL-K). Samples were cultured in enrichment broth and placed in an aerobic incubator at 37 ± 1°C for 18 to 24 hours. The samples were then plated onto ESBL chromogenic agar (CHROMagar, France), cultured aerobically at 37 ± 1°C for 18 to 24 hours, and read for growth of ESBL bacteria. Pink and white colonies were categorized as ESBL-Ec, whereas blue colonies underwent speciation for *K. pneumoniae*, using polymerase chain reaction, to identify ESBL-K isolates. Throughout this article, colonization refers to asymptomatic carriage. The detailed protocol is available at Cocker et al. [14].

This analysis focuses on the Malawian areas: Ndirande (urban), Chileka (periurban), and Chikwawa (rural). We model the presence or absence of colonization with ESBL-Ec and ESBL-K in individuals and aim to detect associations with individual-level demographic and health characteristics, household-level WASH indicators, and the social context represented by the study area. To capture seasonality and household structure, we used a hierarchical multivariable harmonic regression, with temporal correlation at the household level.

### Covariate Selection

To investigate WASH practices, reported variables (ie, presence of a toilet) were collected by asking participants at baseline, whereas observed variables (ie, type of toilet) were answered by field teams observing the household infrastructure. These variables were screened for importance by environmental health specialists (T. M., K. C.) with expertise on the risks and control points for fecal-oral transmission in Malawi [15–17].

Individual- and household-level covariates were also selected. Antibiotic use was defined as the reported use of any antibiotics in the past 6 months (at baseline) and subsequently between each follow-up visit. The survey question was supported by the use of the “drug bag” method and also cross-referenced in the participants’ health passports [18]. Cotrimoxazole prophylactic therapy was excluded to capture only the immediate change expected to occur following short courses of therapy.

Univariable logistic models were used to investigate the effect of WASH infrastructure and associated behaviors on colonization. We started by looking at the effect of the study area on colonization to separate its potential effect from the effects of other risk factors and ran the univariable models accordingly. This allowed for a refinement of the variables to be included in the modeling framework, by retaining them only if *p* <.2. We pragmatically set this threshold to avoid missing the identification of important covariates. A spline model was used to detect nonlinear effects of age, but none was found.

### Multilevel Harmonic Hierarchical Regression

The modeling framework is detailed in Supplementary Methods. Briefly, we used a hierarchical multivariable harmonic logistic regression, incorporating covariates selected by the univariable analysis. We included study region, annual and biannual harmonic terms to capture seasonality, and a serially correlated household-level random effect to reflect temporal correlation in household-level prevalence between timepoints and heterogeneity in prevalence between households over and above that explained by the covariates. The analysis was carried out for both binary colonization response variables. The Bayesian model was fitted using priors [19] and implemented in R v4.1.1 with RStan [20, 21].

Initial model exploration considered adding the random effect at the individual level, but found no evidence of temporal correlation. We therefore applied the serial correlation to the household level, enabling us to quantify both a household effect and to account for possible “household contamination” with an ESBL as a result of a longer term transmission process operating within the home. To ensure maximum identifiability of the random effects, we performed our analysis only on households with sample results for all time points.

Ethical approval was obtained from Liverpool School of Tropical Medicine Research and Ethics Committee, UK (REC, #18-090), and College of Medicine Research and Ethics Committee, Malawi (#P.11/18/2541).

## RESULTS

### Exploratory Analysis

After covariate selection and cleaning, the dataset contained 2493 samples from 259 households (Supplementary Dataset, Supplementary Results). The list of covariates can be found in Supplementary Table 1. The distribution of samples, individuals, and households is presented in Table 1.

**Table 1.** Distribution of the Number of Households, Individuals, and Samples

	Ndirande	Chikwawa	Chileka	Total
Households	96	64	99	259
Individuals	285	259	350	894
Samples	709	891	893	2493

**Figure 1.**
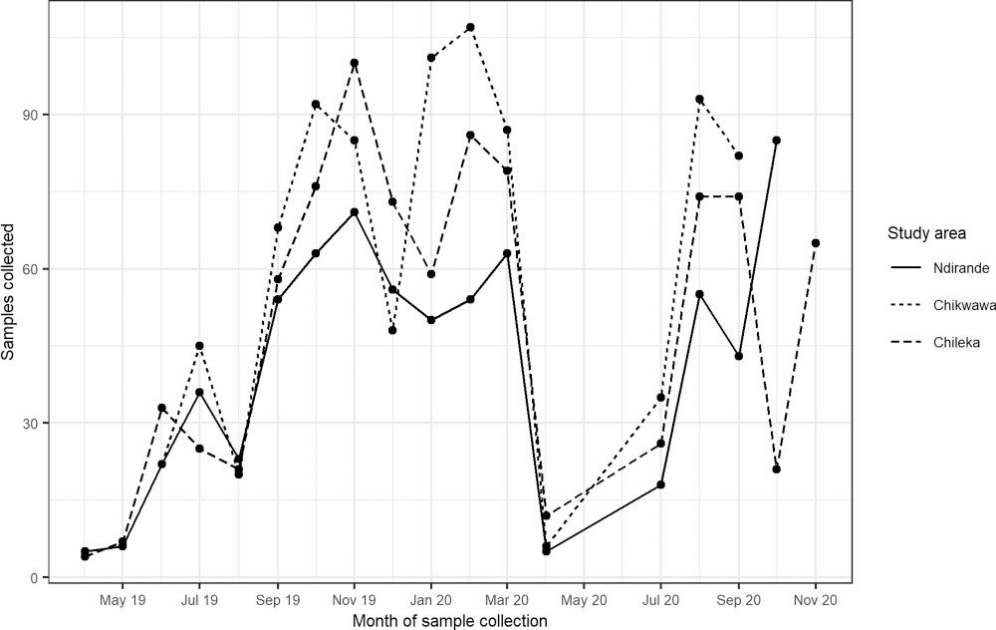
Distribution of samples collected, over time and by study area.

The 129 households for which individuals had 4 available samples were used for the hierarchical model. The distribution of samples over time can be found in Figure 1.

#### Participant Data

Our age distribution data reflect Malawi's population structure. People were considered adults at age ≥16 years (54.0%, 483/894) and school age was ≥5 to 15 years (27.1%, 242/894). A total of 57.1% (510/894) of participants were female. At baseline, 15.2% (129/851) of participants reported having taken at least 1 course of antibiotics, whereas between subsequent visits, 6% (37/616), 9.4% (54/570), and 8.3% (38/456) reported it. This varied by region, with 15.4% (137/891) in Chikwawa, 9.3% (66/709) in Ndirande, and 6.2% (55/893) in Chileka.

**Figure 2.**
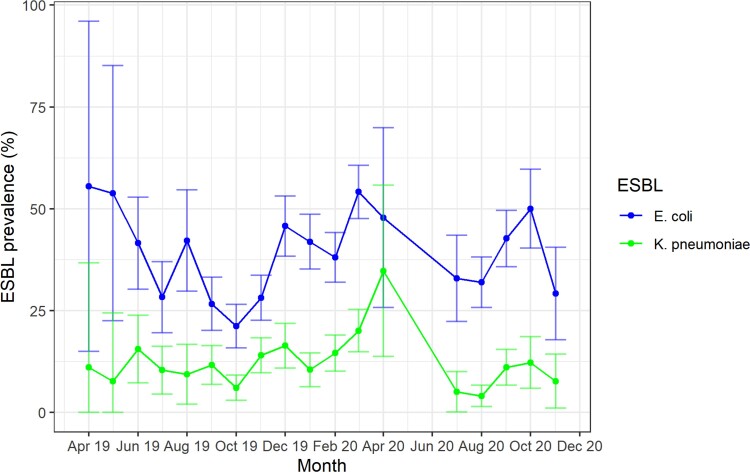
Prevalence of ESBL-producing *E. coli* and ESBL-producing *Klebsiella pneumoniae* per month. Abbreviations: *E. coli*, *Escherichia coli*; ESBL, extended-spectrum β-lactamases.

Overall, the prevalence of ESBL-Ec was 37.0% (922/2493) and the prevalence of ESBL-K was 11.9% (296/2493). Both varied over time, following a similar pattern (Figure 2). Some evidence of seasonality can be discerned for both, especially for *E. coli*, with a decrease in prevalence during the dry season (May–October) followed by an increase during the wet season (November–April).

#### Household Data

More than 75% (202/259) of households had between 3 and 6 members, with a median of 4 individuals. Nine percent (24/259) had 2 individuals, whereas 13% (33/259) had more than 6 individuals. More than 50% (156/259) had a monthly income between 15 000 and 50 000 Malawi Kwacha (mwk). Thirty-eight households had less than 15 000 mwk, whereas 65 households had more than 50 000 mwk. The median income was 30 000 mwk.

#### WASH Data

**Figure 3.**
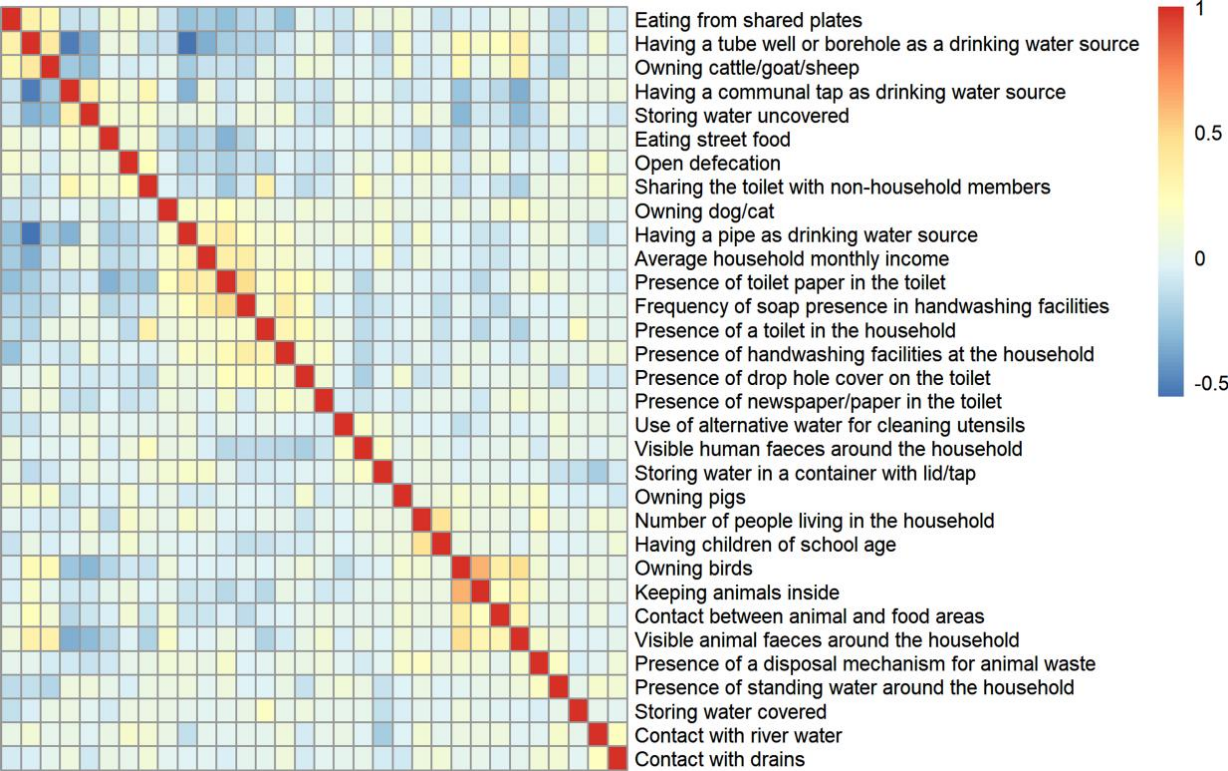
Correlation heatmap of household-level covariates. Values range from −0.5 (blue) to 1 (red) and represent the correlation coefficient between every pair of household-level covariates. Covariates are in the same order on rows and columns.

Correlations between household-level variables are depicted in Figure 3. There was a positive correlation between animal exposure–related factors (eg, bird owners appeared more likely to keep animals inside, which were more likely to come into contact with food preparation areas). With increasing income, there was increasing likelihood the household's water drinking source came from a pipe, rather than a tube well or borehole. Moreover, increasing income correlated with presence of hand washing facilities and soap in the household, and of cleaning materials near the toilet. Eating from shared plates appeared to be negatively correlated with these sanitation factors; the higher the household income, the less chance individuals used shared plates.

### Impact of WASH Infrastructure and Associated Behaviors on ESBL Colonization

To start exploring the effect of WASH variables on ESBL colonization, and to look at variability between regions, we first ran a generalized linear model including only the study area. There was a higher risk of being colonized in Ndirande (odds ratio [OR], 1.39; confidence interval, 1.13–1.70; *P* = .002) compared with Chileka. No significant effect was detected for ESBL-K. Consequently, univariable analysis was run with the study area included as a covariate for ESBL-Ec, but not for ESBL-K.

#### Human Gut Mucosal Colonization With ESBL-producing *E. coli*

Results from the univariable models showed that having a drinking source coming from a tube well or a borehole, having a drop hole cover on the toilet, and allowing animal contact with food areas appeared to be highly significant. Although a positive association with colonization was detected for the source coming from a tube well or a borehole, the opposite can be said for piped water. Animal contact with food areas, keeping animals inside, and having a toilet floor surfaced with soil were significant and positively associated with colonization. Having a drop hole cover and having clean paper in the toilet were also significant but appeared to have a protective effect. Older age, visible open defecation in the area, owning cattle, sheep, or goats, and contact with river water were significant (<0.05) and positively associated with colonization. Male sex, higher income, having a disposal mechanism for animal waste, having a piped water drinking source, storing water in a container with lid and tap were negatively associated with ESBL-Ec colonization (Supplementary Table 2).

The hierarchical model showed that men were less at risk of becoming colonized with ESBL-Ec (OR, 0.786; credible interval [CrI], .678–.910) and that having a tube well or a borehole as a drinking source increased your risk (OR, 1.550; CrI, 1.003–2.394). Coming into contact with standing water appeared to be negatively associated with colonization (OR, 0.749; CrI, .574–.978). Finally, an apparent signal of annual seasonality was noticeable from the presence of part of the harmonic term (Table 2).

**Table 2.** Temporal Model Results for ESBL-producing *E.coli* Colonization Status

	Log-odds	Odds Ratio (95% CrI)
(Intercept)	−0.716	0.489 (.360–.663)
Reactive to HIV testing (vs nonreactive)	0.027	1.027 (.863–1.223)
Unknown HIV status (vs nonreactive)	−0.031	0.969 (.808–1.163)
Recent use of antibiotics	0.093	1.097 (.946–1.274)
Age	0.132	1.141 (.970–1.343)
Being male (vs female)^a^	−0.241	0.786 (.678–.910)
Average household monthly income	0.226	1.254 (.916–1.715)
Open defecation	0.054	1.055 (.826–1.349)
Presence of a disposal mechanism for animal waste	0.104	1.110 (.857–1.437)
Eating from shared plates	−0.245	0.783 (.598–1.024)
Having a pipe as drinking water source	0.132	1.141 (.818–1.592)
Having a tube well/borehole as drinking water source^a^	0.438	1.550 (1.003–2.394)
Use of alternative water for cleaning utensils	0.014	1.014 (.802–1.283)
Owning cattle, goats, or sheep	0.139	1.149 (.892–1.480)
Keeping animals inside	0.075	1.078 (.852–1.364)
Contact with river water	0.048	1.049 (.791–1.391)
Toilet floor material: none (vs concrete/wood)	0.123	1.131 (.799–1.600)
Toilet floor material: soil (vs concrete/wood)	0.131	1.140 (.820–1.585)
Presence of drop hole cover on the toilet	−0.202	0.817 (.626–1.067)
Presence of newspaper/paper in the toilet	−0.155	0.856 (.670–1.094)
Frequency of soap presence in handwashing facilities	−0.000	1.000 (.640–1.563)
Storing water covered	−0.179	0.836 (.537–1.302)
Storing water in a container with lid/tap	−0.034	0.967 (.754–1.240)
Contact between animal and food areas	0.218	1.244 (.983–1.573)
Presence of standing water around the household^a^	−0.289	0.749 (.574–.978)
Number of days since the first sample	0.167	1.182 (.869–1.608)
Harmonic term (sinday)^a^	−0.466	0.628 (.453–.869)
Harmonic term (cosday)	0.371	1.449 (.958–2.191)
Harmonic term (sinday2)	−0.084	0.919 (.644–1.314)
Harmonic term (cosday2)	0.018	1.018 (.711–1.457)
Living in Chikwawa (vs Chileka)	−0.276	0.759 (.527–1.093)
Living in Ndirande (vs Chileka)	0.386	1.471 (.980–2.207)

Abbreviations: CrI, credible interval; *E. coli*, *Escherichia coli*; ESBL, extended-spectrum β-lactamases; HIV, human immunodeficiency virus.

Significant variables.

Using the covariance structure (Supplementary Methods), the range of temporal correlation was estimated at 77.85 days (CrI, 30.85–140.60); thus, samples from the same household obtained more than 77 days apart are effectively uncorrelated, meaning that an individual's ESBL status at 78 days is not influenced by the status at baseline (Supplementary Results).

#### Human Gut Mucosal Colonization With ESBL-producing *K. pneumoniae*

Univariable models showed that household size was highly significant (*P* < .01), revealing a greater risk of being colonized with increasing household size. Eating street food and eating from shared plates appeared to have a significant protective effect on colonization. Contrarily, owning birds and coming into contact with drains were significant and positively associated with colonization (Supplementary Table 3).

The hierarchical model for ESBL-K found that antibiotic use increased the risk of being colonized (OR, 1.281; CrI, 1.049–1.565). Eating from shared plates was negatively associated with colonization (OR, 0.672; CrI, .460–.980). Finally, a signal of annual seasonality was also detected (Table 3). The range of temporal correlation for ESBL-K colonization was estimated at 54.29 days (CrI, 12.91–130.43).

**Table 3.** Temporal Model Results for ESBL-producing *Klebsiella pneumoniae* Colonization Status

	Log-odds	Odds Ratio (95% CrI)
(Intercept)	−3.432	0.032 (.017–.060)
Recent use of antibiotics^a^	0.248	1.281 (1.049–1.565)
Number of people living in the household	0.298	1.347 (.932–1.947)
Presence of a toilet in the household	−0.136	0.873 (.468–1.628)
Eating street food	0.091	1.095 (.797–1.505)
Eating from shared plates^a^	−0.398	0.672 (.460–.980)
Having a pipe as drinking water source	−0.253	0.776 (.506–1.190)
Having a communal tap as drinking water source	−0.423	0.655 (.408–1.051)
Use of alternative water for cleaning utensils	−0.241	0.786 (.563–1.097)
Owning birds	0.015	1.015 (.706–1.459)
Owning dogs or cats	0.024	1.024 (.735–1.427)
Owning pigs	0.157	1.170 (.853–1.604)
Contact with drains	0.219	1.245 (.906–1.710)
Toilet type: other (vs no toilet)	0.203	1.225 (.752–1.996)
Toilet type: pit latrine (vs no toilet)	0.204	1.226 (.550–2.734)
Toilet type: shared toilet (vs no toilet)	−0.395	0.674 (.395–1.148)
Visible human faeces around the household	0.224	1.251 (.881–1.777)
Storing water uncovered	−0.326	0.722 (.478–1.089)
Number of days since the first sample	−0.066	0.936 (.587–1.493)
Harmonic term (sinday)^a^	−0.753	0.471 (.289–.767)
Harmonic term (cosday)	0.448	1.565 (.883–2.774)
Harmonic term (sinday2)	−0.304	0.738 (.434–1.255)
Harmonic term (cosday2)	0.483	1.621 (.961–2.736)
Living in Chikwawa (vs Chileka)	−0.038	0.963 (.606–1.529)
Living in Ndirande (vs Chileka)	0.274	1.315 (.812–2.130)

Abbreviations: CrI, credible interval; ESBL, extended-spectrum β-lactamases.

Significant variables.

## DISCUSSION

This study identified varying prevalence of ESBL colonization over time, with higher prevalence during the wet season. Potential explanations include the accumulation of mud and floodwater, which might lead to more contact with contaminated soil or water. Additionally, the increased time spent indoors during periods of heavy rain might lead to higher within-household transmission. The prevalence is consistent with previous studies on ESBL colonization in sSA [5, 6, 22]. However, although most of these studies were from a specific subset of the population, our study focused on the general population in community settings. Comparing with community studies exclusively, we found a prevalence of 37%, which is much higher than the previous estimate of 18% (95% confidence interval, 11%–28%) for community members in sSA [5]. This prevalence is close to some of the highest reported in the world [6].

The correlation heatmap (Figure 3) suggested that the socioeconomic status of the household influences the household WASH situation, and that higher income allows for a better access to cleaner water and sanitation and hygiene products. Being female was identified as a risk factor for ESBL-Ec, perhaps because women are more likely to perform domestic duties (ie, laundry, housework, and childcare), which would place them at higher risk of being in contact with the fecally contaminated environment. However, no direct association was found between income and colonization here, perhaps because the vast majority of households in the study were below the threshold of absolute poverty and that income alone is a poor indicator of wealth.

Study area, keeping animals inside, and animal contact with food preparation areas also conferred an increased risk for colonization with ESBL-Ec in the univariable models. These animal husbandry practices are common in sSA [23]; however, they put household members at higher risk of exposure to fecal pathogens and enteric infections [24, 25]. Having access to cleaning materials and a drop hole cover were negatively associated with colonization. The latter being used to prevent flies from accessing fecal matter, this association is consistent with studies showing the role of flies in transporting and transmitting *E. coli* [26, 27].

Eating from shared plates and the presence of standing water around the household appeared to have a protective effect. This is a surprising result because plate-sharing, which is common in low- and middle-income countries [28], has been associated with transmission of enteric pathogens in other settings [29]. Similarly, because wastewater is known to play a role in the transmission of AMR [12], both would seem to promote transmission. As with all statistical inference, we caution that these results might represent at type I error (in a Bayesian context), and that further research into WASH behavioral patterns and interactions with other explanatory variables would be necessary to confirm our findings.

Antibiotic use was identified as a risk factor for ESBL-K, consistent with previous studies in sSA [5, 8–10]. In Malawi, antibiotics are widely available by formal and informal routes [30]. Lack of healthcare workers at many facilities results in day-long waiting times for patients, driving them to search for alternative ways to access antimicrobials, usually without consultation or prescription. Additionally, to alleviate waiting times and accommodate the high volume of patients, workers may dispense antimicrobials directly to patients without a prescription [31]. Therefore, the identification of antibiotic use as a risk factor may also reflect recent healthcare exposure. The reason for the variation between study areas is uncertain. In Chikwawa, the rural area of the study [14], participants are likely to have greater exposure to livestock, and may have had increased access to antibiotics through nongovernmental organizations [32]. This emphasizes the importance of antimicrobial exposure in driving ESBL colonization, thus highlighting the need for optimizing community antibiotic usage.

For ESBL-K, at the univariable level, household size was the only highly significant risk factor, highlighting the importance of the household in driving ESBL transmission. Other significant variables included owning birds, which are known to be responsible for fecal contamination of the household environment in low- and middle-income countries [33], and contact with drains, highlighting again the importance of interactions among animals, humans, and environment.

Temporal models detected a temporal correlation range of 8 to 11 weeks. In other words, 2 samples taken within that time frame are more likely to both be colonized than if spread apart any further in time. Though our method was designed only to detect association between ESBL prevalence in subsequent follow-ups, it does suggest that within-household transmission occurs within this time frame. Subsequent causal inference studies would be required to confirm this. An understanding of how long colonization lasts after acquisition is needed to tailor appropriate interventions.

This study had some limitations. Variables had to be preselected based on perceived importance. We could not find temporal correlation at the individual level, which suggested that an individual's samples could be seen as independent from each other. Potential explanations include the use of stool samples over rectal swabs, which may have been better for screening. Additionally, the laboratory testing protocol was qualitative, discriminating only between presence or absence of ESBLs. Further work is needed to consider the impact of microbiological methods on informing these models. Whole genome sequencing will allow a thorough investigation of the linkage between sequence types. The coronavirus disease 2019 pandemic caused the sampling and microbiological testing to be suspended, causing some delay in our data collection (April–July 2020).

Our study suggests that WASH factors and environmental hygiene are key drivers of AMR transmission in Malawi, consistent with findings in other African settings [34]. Our results also point toward acquisition of ESBL-producing *E. coli* through contaminated water and/or inappropriate WASH infrastructure. Additionally, seasonality and gender also suggest the importance of environmental hygiene and practices in driving ESBL-producing *E. coli* transmission. This underlines the need for improved access to clean water and suggests that associating WASH behavioral practice with better WASH conditions would be instrumental in decreasing transmission. However, for ESBL-producing *K. pneumoniae*, antibiotic use was identified as a risk factor, therefore emphasizing the importance of antimicrobial exposure in driving transmission and the need for improved infection prevention and control measures and antibiotic usage and stewardship training. A better understanding of how the WASH factors of the different communities impact ESBL colonization and transmission will inform public health responses to the challenge presented by AMR and enable the design of effective intervention strategies in sSA.

## Supplementary Data

Supplementary materials are available at *Clinical Infectious Diseases* online. Consisting of data provided by the authors to benefit the reader, the posted materials are not copyedited and are the sole responsibility of the authors, so questions or comments should be addressed to the corresponding author.

## Supplementary Material

ciad117_Supplementary_Data

## Notes

***Acknowledgments.*** The authors thank the participants for taking part in the study, as well as the wider DRUM consortium (https://www.drumconsortium.org/about/the-drum-team) for their advice and support. They thank Lancaster University for the use of their High End Computer, and in particular Dr Mike Pacey for his help in setting up their software stack.

For the purpose of open access, the author has applied a CC BY public copyright licence (where permitted by UKRI, “Open Government License” or “CC BY-ND public copyright license” may be stated instead) to any Author Accepted Manuscript version arising.

***Financial support.*** This work was funded through a grant awarded to N. F. and S. T. J. [MR/S004793/1] from the Medical Research Council, a Council of UK Research and Innovation (https://www.ukri.org/), and the National Institute for Health Research (https://www.nihr.ac.uk/). The funders had no role in study design, data collection and analysis, decision to publish or preparation of the manuscript.

***Data availability statement.*** The authors confirm that the data supporting the findings of this study are available within its Supplementary Materials.
