# Supplementary material for: Risk Factors, Temporal Dependence, and Seasonality of Human Extended-Spectrum β-Lactamases-Producing Escherichia coli and Klebsiella pneumoniae Colonization in Malawi: A Longitudinal Model-Based Approach
Source: Clin Infect Dis. 2023 Mar 4;77(1):1–8. doi: 10.1093/cid/ciad117 (PMC10320086; doi:10.1093/cid/ciad117)
Supplement: ciad117_Supplementary_Data [file ciad117_supplementary_data.zip › Supplementary Results.docx]

**Exploratory analysis**

Household, individual and WASH datasets were merged to form a unique dataset, removing incomplete and duplicate records assuming missingness at random. Twenty-seven households were excluded due to missing enrolment or WASH data. Four individuals were removed due to missing data on age or gender, eighteen duplicate records were removed. Twenty-six WASH variables were selected for further analysis and three were removed due to a lack of variation. Out of 2845 human stool samples collected by field teams over time, forty-four duplicate records were removed. After merging, 224 samples were removed due to missing covariate data, and 84 samples from households≥200 meters outside of the polygon limits, were also removed. This threshold permitted retention of households subsequently chosen by the field teams after refusal from the original sampled household. The variables were standardised, therefore odds ratio should be interpreted as a change for each increase in standard deviation.

**Parameter estimates and prior and posterior densities**

Parameter estimates are shown below in Supplementary Table a. The densities of the priors and posteriors of all three parameters can be found in Supplementary Figure a. Visual inspection of the trace plots in Supplementary Figure b and calculations of the Gelman-Rubin statistic resulting close to 1 for all parameter estimates indicates that the model has fitted properly.

**Supplementary Table a. Estimates for φ, σ and τ in the ESBL-*E. coli* temporal model.**

|  | **Estimate (95% CrI)** | **Standard deviation** |
| --- | --- | --- |
| **Phi φ** | 45.28 (18.82-81.36) | 15.87 |
| **Sigma σ** | 1.25 (0.57-1.73) | 0.29 |
| **Tau τ** | 1.29 (0.89-1.69) | 0.20 |


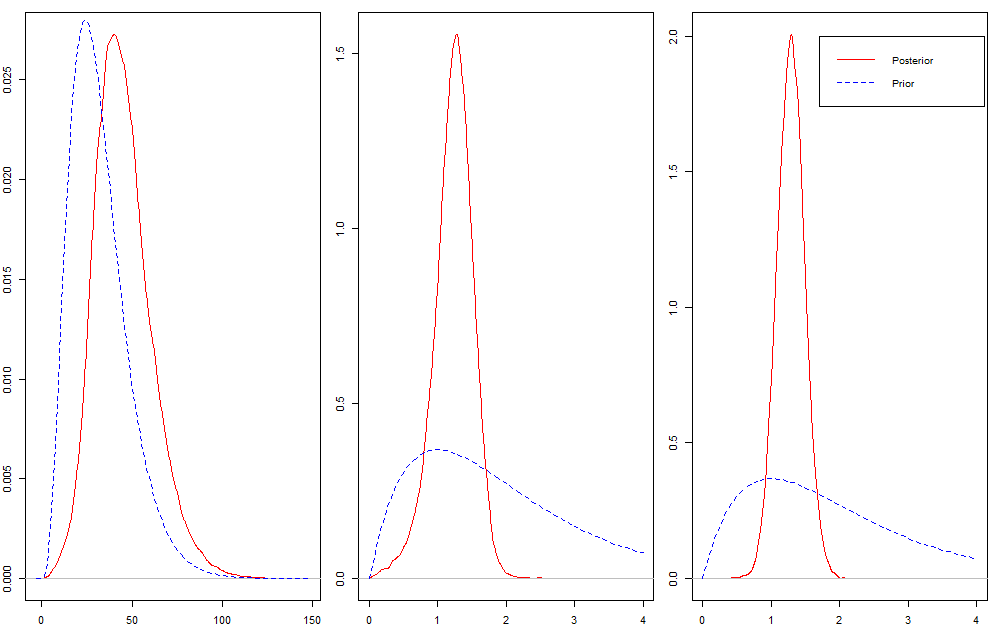


**Supplementary Figure a. Prior and posterior density of φ, σ and τ (left to right, without warm-up) for the ESBL *E. coli* temporal model.**


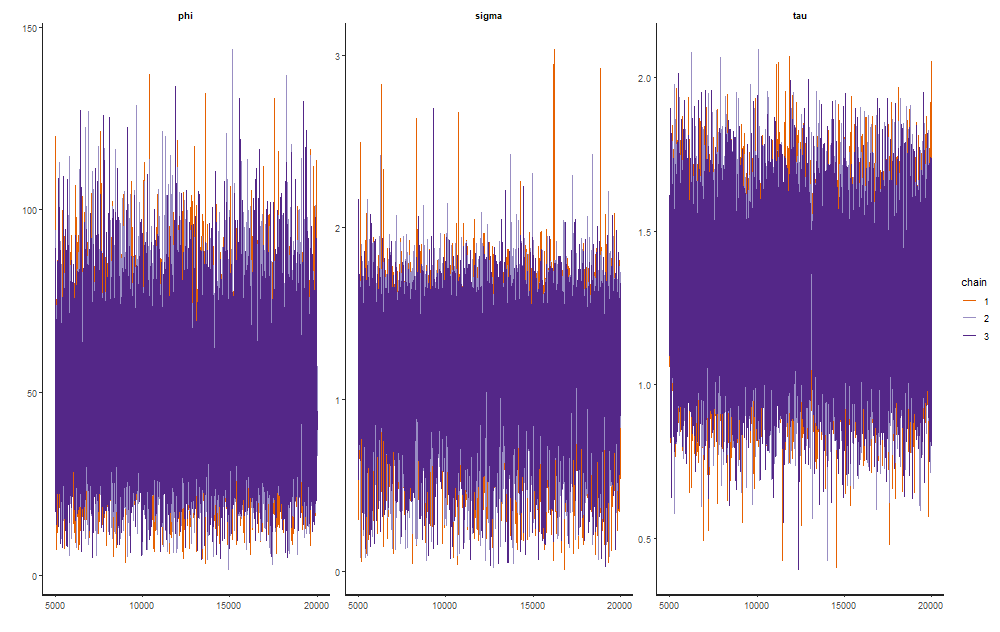


**Supplementary Figure b. Trace plots of φ, σ and τ (left to right, without warm-up) for the temporal model for ESBL *E. coli.***

Parameter estimates are shown in Supplementary Table b. The densities of the priors and posteriors of all three parameters can be found in Supplementary Figure c. Convergence was verified by looking at the trace plots in Supplementary Figure d and we confirmed that the Gelman-Rubin statistic was close to 1 for all parameter estimates.

**Supplementary Table b. Estimates for φ, σ and τ in the ESBL-*K. pneumoniae* temporal model.**

|  | **Estimate (CrI 95%)** | **Standard deviation** |
| --- | --- | --- |
| **Phi φ** | 31.54 (7.78-75.21) | 17.91 |
| **Sigma σ** | 1.17 (0.24-2.79) | 0.66 |
| **Tau τ** | 1.63 (1.05-2.28) | 0.31 |


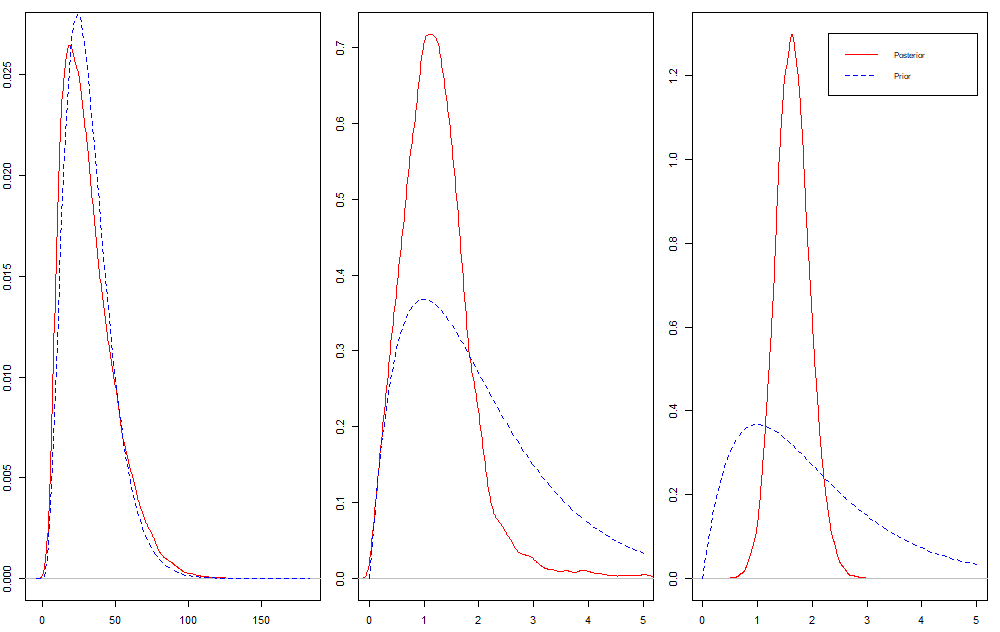


**Supplementary Figure c. Prior and posterior density of φ, σ and τ (left to right, without warm-up) for the ESBL *K. pneumoniae* temporal model.**


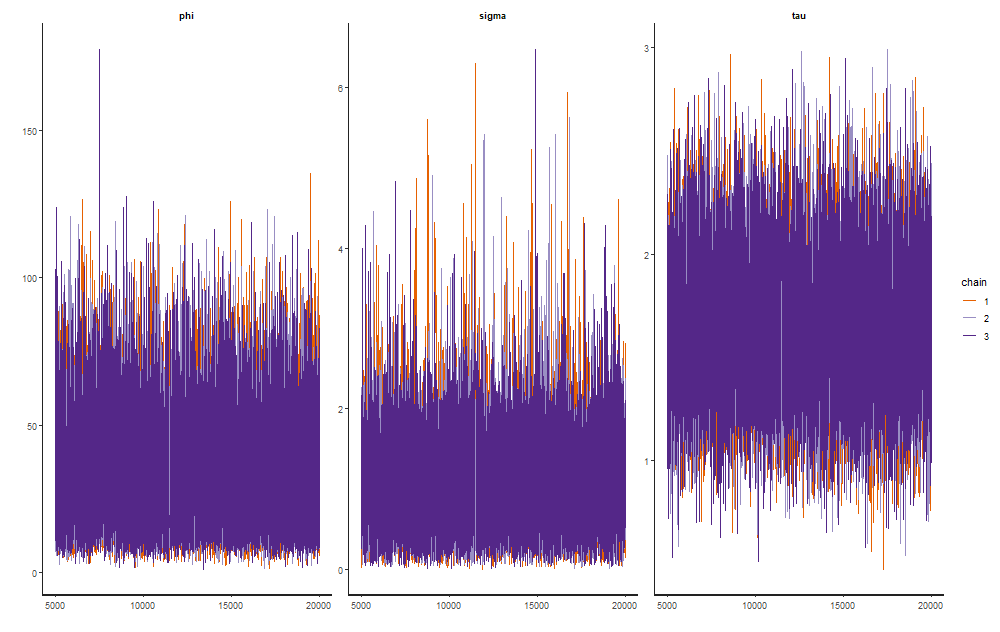


**Supplementary Figure d. Trace plots of φ, σ and τ (left to right, without warm-up) for the temporal model for ESBL *K. pneumoniae.***
